# Treadmill Exercise Stress Test-Induced Takotsubo Cardiomyopathy: A Case Report and Review of Literature

**DOI:** 10.7759/cureus.33426

**Published:** 2023-01-05

**Authors:** Annapoorna Singh, Daulath Singh

**Affiliations:** 1 Internal Medicine, University of Kansas Health System, St. Francis Hospital, Topeka, USA; 2 Internal Medicine, Hematology-Oncology, Stormont Vail Healthcare, Topeka, USA

**Keywords:** exertional chest pain, myocardial ischemia, trademill exercise test, takotsubo cardiomyopathy, stress ind cardiomyopathy

## Abstract

Noninvasive stress testing is routinely indicated and preferable in the diagnosis of coronary artery disease. We present the case of a patient who developed Takotsubo syndrome/cardiomyopathy (TTS) as a result of an exercise stress echocardiography, as well as a literature review of comparable cases. An abnormal stress test necessitated coronary angiography, which revealed nonobstructive coronaries with apical left ventricular ballooning and a decreased ejection fraction (EF), both of which are concerning for TTS. The patient was medically managed with metoprolol and lisinopril, with improvement in the EF on the follow-up echocardiogram.

## Introduction

Coronary artery disease (CAD) remains the leading cause of mortality in the United States and across the world [1]. Noninvasive diagnostic testing (either exercise or pharmacological) is preferred and plays an important role in the diagnosis of cardiovascular disorders [2]. In addition to providing predictive value [2], the exercise stress test is inexpensive, widely accessible, and reasonably safe [3]. In this case report, we highlight the case of a patient who experienced Takotsubo syndrome/cardiomyopathy (TTS) during an exercise stress echocardiography.

## Case presentation

A 62-year-old Caucasian woman with no significant past medical history presented to the office for evaluation of her chest pain, which had been ongoing for about six months. The chest pain was retrosternal, worse with exertion, and associated with shortness of breath. The patient endorsed some significant stressors in her life for the preceding six months. The physical examination was unremarkable. The jugular venous pressure was normal, and the cardiac sounds were normal with no murmurs.

Investigations

**Figure 1:**
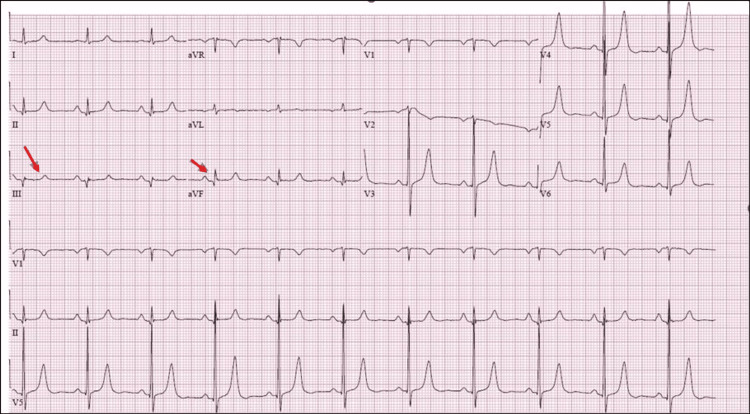
EKG showing sinus rhythm and possible inferior infarct. ST-segment elevation in the inferior lead (red arrows).

EKG was notable for a normal sinus rhythm with criteria for left ventricular (LV) hypertrophy and a possible inferior infarct (Figure 1).

**Figure 2:**
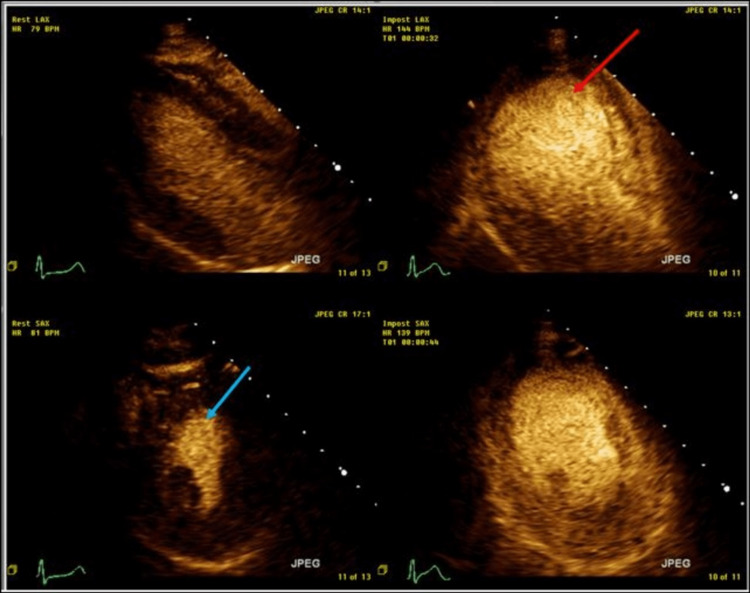
Echocardiographic images of the left ventricle at rest and during stress. Echocardiographic images of the left ventricle demonstrate adequate thickening and contraction of the myocardium at rest (blue arrow) as compared to exercise-induced global dilatation and apical ballooning of the left ventricle at stress (red arrow).

The baseline echocardiogram (echo) showed normal LV systolic function with an ejection fraction (EF) of 60% and concentric LV hypertrophy. She subsequently had an exercise stress echocardiogram. At the peak of exercise, the LV appeared dilated with global hypokinesis and an EF of about 25% (Figure 2).

Along with this, the patient also had chest pain, which resolved by the end of recovery.

Management

**Figure 3:**
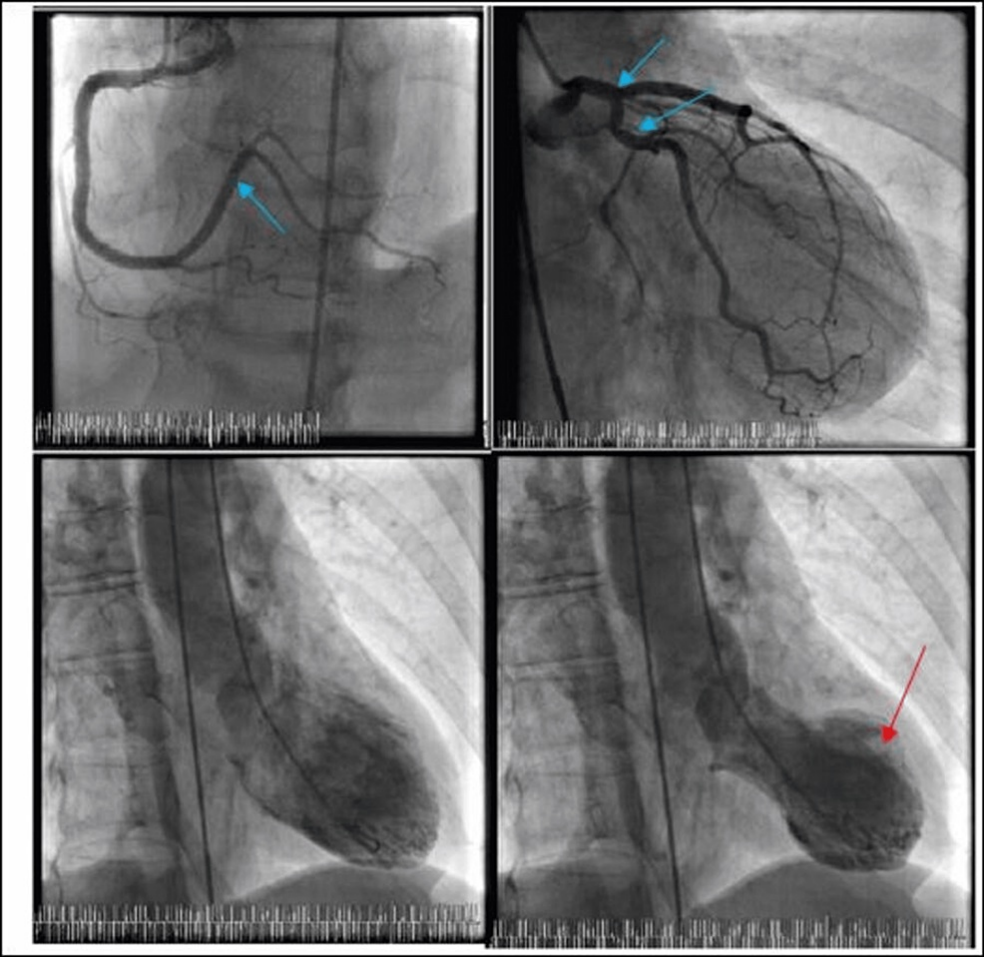
Coronary angiogram with left ventriculogram demonstrating nonobstructive coronary artery disease (blue arrows), apical ballooning and Takotsubo-appearing left ventricle (red arrow), respectively.

The abnormal stress test prompted a diagnostic angiogram, which revealed nonobstructive coronary artery disease (CAD). The left ventriculogram revealed an EF of about 25% with a Takotsubo-appearing LV with apical ballooning and basilar hyperkinesis (Figure 3).

The abnormal tests were thus attributed to the exercise portion of the stress test, as her baseline echocardiogram was normal. There was no clinical suspicion for other inciting factors, such as pheochromocytoma and myopericarditis, and therefore, an exhaustive workup was not pursued. The patient was prescribed metoprolol and lisinopril for stress cardiomyopathy.

Follow-up

The patient had a repeat echo two months following the diagnosis of TTS, which showed complete recovery of LV function with an EF of 60-65% and normal wall motion. 

## Discussion

First described in 1990 in the Japanese population, Takotsubo cardiomyopathy or stress cardiomyopathy can be caused by various inciting factors. Most common among these are emotional triggers, physical activities, and neurological illness, as well as other medical conditions and procedures. The diagnosis is based on the Mayo Clinic criteria [4].

Although previously thought to be a benign condition, it has now come to light that patient with TTS carries a similar short-term and long-term mortality as those of age- and sex-matched patients with acute coronary syndrome (ACS). The prognosis depends on the inciting stress factors, such that those with physical activities and medical conditions or procedures as the inciting factors have the worst long-term outcomes, while those with emotional triggers have relatively better long-term outcomes [5]. Among the various physical stressors, exercise has been described in the literature as a relatively uncommon etiology of TTS [6]. Here, we review all reported cases and clinical features of exercise-induced TTS available in the literature (Table 1)

**Table 1:** Review of the reported cases and clinical features of exercise-induced Takotsubo cardiomyopathy available in the literature. DES: dobutamine stress echocardiogram; EF: ejection fraction; METS: metabolic equivalents; MHR: maximum heart rate; SPECT: single photon emission computerized tomography; WMA: wall motion abnormality.

Study	Age	Sex	WMA and EF	Symptom	EKG changes		Stress intensity	Troponin	Coronary angiogram results	Follow-up
Magri et al. [7]	69	Female	Apical akinesis, anterior hypokinesis, and mild impairment of global systolic function	Chest pain, emotional stressors	ST elevations in I, aVL, V2, V3 and ST depression in III, aVF, V4-V6	Three METS		9.7 ng/mL	Normal	Complete resolution of echo findings after one month
Irwin et al. [8]	81	Female	Akinesis of midventricular and apical segments. Sparing of the base. EF 38%	Chest pain	ST elevation in the anterolateral leads 15 min post-exercise	89% of MHR		0.86 µg/L	Mild diffuse atherosclerosis	Improvement in mid-apical systolic function after seven days; EF 56%
Dhoble et al. [9] (case no. 1)	50	Female	Apical akinesis and mid-ventricular hypokinesis; EF 50%	Chest pain	< 1 mm J point elevation in II, III, and aVF	7.2 METS		0.22 UI/L	Normal	A dobutamine stress echocardiogram (DES) performed three days later was normal, with EF of 70%
Dhoble et al. [9] (case no. 2)	75	Male	Akinetic basal segment with normal apical segments; EF 55%	Pre-operative evaluation for carotid stenosis	ST-elevation	4.1 METS		0.78 UI/L	Normal	DES performed the next day was normal, with EF of 70%
Digne et al. [10]	66	Female	Akinesis of the septo-apical region	Chest pain and emotional stress	Biphasic T waves from V1-V5	85% of MHR		0.22 UI/L	Normal	Complete resolution after two weeks; EF of 60%
López-Cuenca et al. [11]	53	Female	Midventricular, apical WMA; EF 45-50%	Chest pain and dyspnea	T wave depression	63% of MHR		0.19 ng/mL	Normal	Complete resolution; EF of 65%
Dorfman et al. [6]	71	Female	Apical dyskinesis on SPECT	Chest pain	ST depression in V5 and V6	Target heart rate achieved		0.23 UI/L	Normal	Complete resolution on echo one month later
Cantor et al. [12]	77	Female	Mid-cavitary hypokinesis with basal and apical hyperkinesis	Palpitations	ST elevations in I, aVL, V5, V6; ST depression in III, aVF, and V1-V3	4.5 METS		11.17 ng/mL	Nonobstructive CAD	Resolution of WMA on echo obtained two weeks later

**Figure 4:**
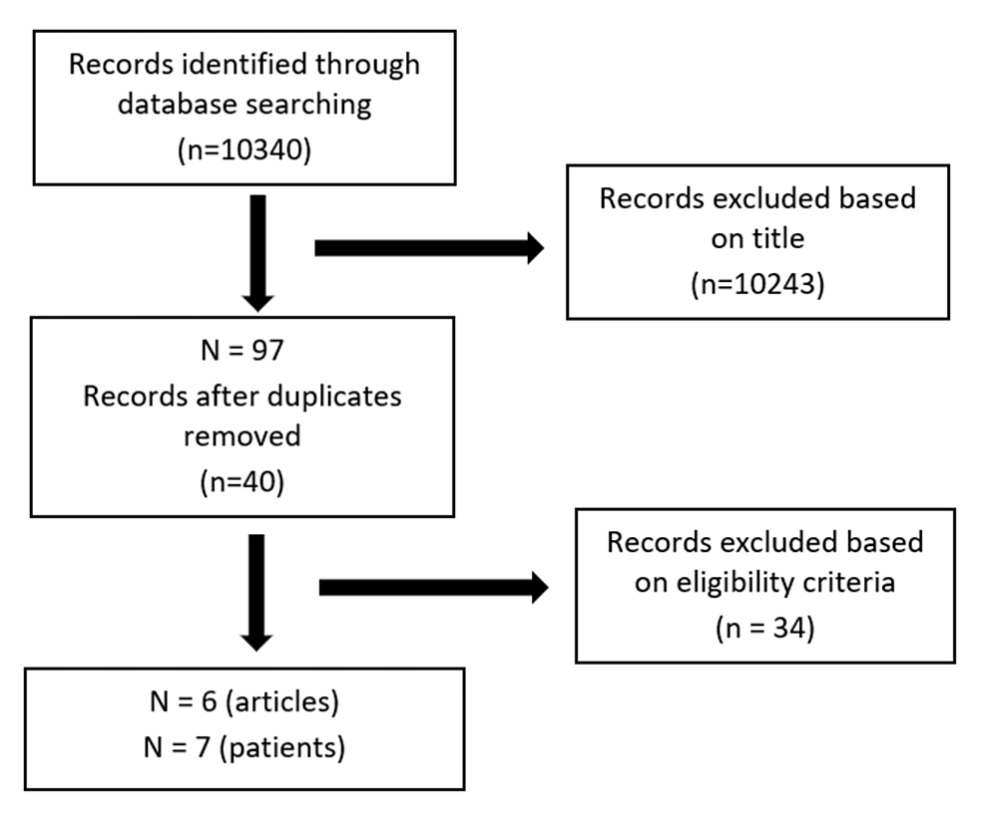
The inclusion and exclusion process of the studies per the criteria. Eligibility criteria for study selection include (1) development of TTS after the exercise stress test, (2) absence of other known comorbidities that can cause cardiomyopathy, and (3) articles in the English language.

We searched MEDLINE (via PubMed) and Google Scholar up to August 12, 2018, and all the studies imported are shown in Figure 4.

Our review reveals that exercise stress test-induced TTS is indeed a real phenomenon. TTS caused by other physically strenuous activities has also been reported in the literature [13]. As mentioned earlier, since TTS associated with physical activities is associated with similar short-term and long-term outcomes [14] as in age- and sex-matched controls with ACS, the findings of TTS associated with exercise stress tests could potentially carry prognostic information [15]. Conversely, to avoid false positive exercise stress echocardiograms, consideration can be made toward obtaining another form of ischemic evaluation such as a regadenoson myocardial perfusion imaging, especially in specific populations such as the post-menopausal woman with multiple stressors in life who are predisposed to TTS [16].

## Conclusions

Takotsubo cardiomyopathy can sometimes be precipitated by exercise stress tests. The presenting symptoms and ECG abnormalities are very similar to those of typical TTS (chest pain and ST-segment elevation). Clinicians must be aware of this risk during the examination. As TTS can be caused by the exercise element of a stress test, individuals who are susceptible to TTS may benefit from an alternative stress modality.
